# Diagnostic accuracy of ICD code versus discharge summary-based query for endocarditis cohort identification

**DOI:** 10.1097/MD.0000000000028354

**Published:** 2021-12-23

**Authors:** H. Nina Kim, Ayushi Gupta, Kristine Lan, Jenell Stewart, Shireesha Dhanireddy, Maria A. Corcorran

**Affiliations:** Department of Medicine, University of Washington, Seattle, Washington.

**Keywords:** discharge summary, endocarditis, ICD code

## Abstract

Studies of infective endocarditis (IE) have relied on International Classification of Disease (ICD) codes to identify cases, a method vulnerable to misclassification. Clinical narrative data could offer greater accuracy and richness to cohort identification. We evaluated two algorithms:

a standard query of ICD-9/10 billing codes, with or without procedure codes for echocardiogram and

a text query of discharge summaries (DS) that selected on the term “endocarditis” in fields headed by “Discharge Diagnosis” or “Admission Diagnosis” or similar.

Further coding extracted valve involved and organism responsible if present. All cases were chart reviewed using pre-specified criteria. Positive predictive value (PPV), sensitivity and specificity were calculated. The ICD-based query identified 612 individuals from July 2015 to July 2019 who had a hospital billing code for infective endocarditis; of these, 534 had an echocardiogram. The DS query identified 387 cases. PPV for the DS query was 84.5% (95% CI 80.6%, 87.8%) compared with 72.4% (95% CI 68.7%, 75.8%) for ICD only (*P* < .001) and 75.8% (95% CI 72.0%, 79.3%) for ICD + echo queries (*P* = .002). Sensitivity was 75.9% for DS query and 86.8% to 93.4% for ICD queries (*P* < .02 for these comparisons). Specificity was high for all queries >94%. The DS query also yielded valve data (prosthetic, tricuspid, aortic, etc) in 60% and microbiologic agent in 73% of identified cases with an accuracy of 94% and 90%, respectively when assessed by chart review. Compared with ICD-based queries, text-based queries of discharge summaries have the potential to improve precision of IE case ascertainment and extract key clinical variables.

## 1 Introduction

Even in the modern antibiotic era, infective endocarditis (IE) is associated with a 1-year mortality in the range of 20% to 30%.^[1,2]^ The incidence of IE is increasing in the United States, particularly among younger individuals and those who inject drugs.^[3–5]^ Gaining a contemporary understanding of this severe infection is a priority and requires accurate cohort identification. Observational studies to date have relied heavily on International Classification of Disease (ICD) diagnosis codes to extract IE cases.^[3–6]^ This method, while highly time-efficient, may be prone to misclassification due to coding inaccuracies.^[7–9]^ ICD coding for hospital care is often not performed by clinicians but rather professional coders^[10]^ and can be inconsistent across clinical institutions.^[11]^ Use of ICD codes without verification of event accuracy is a common practice, and few studies have validated ICD codes for IE.^[12–15]^

Discrimination between historical and current clinical events is critical for patient identification for clinical registries and outcomes research but may be suboptimal with ICD codes.^[16]^ Additionally, key clinical characteristics of IE, such as valvular features or microbiologic data, are often not appropriately captured in ICD codes, or captured at all. Examination of clinical narrative data, specifically discharge summaries, could offer greater accuracy and richness to efforts to define a clinical cohort.^[17,18]^ The aim of this study was to evaluate the comparative performance characteristics—positive predictive value, sensitivity, and specificity—of two algorithms for IE case identification:

1. ICD codes with or without a procedure code for echocardiogram and
2. a rule-based keyword search of discharge summaries to identify patients hospitalized and newly diagnosed with infective endocarditis.

## 2 Methods

We conducted a retrospective methodological research study of diagnostic accuracy of two methods of IE cohort identification. We identified patients who were discharged from one of two academic teaching hospitals in Seattle, Washington any time from July 1, 2015 to July 31, 2019:

1. University of Washington Medical Center (UWMC), a 570-bed tertiary/quaternary care facility and
2. Harborview Medical Center (HMC), a 413-bed acute care hospital that serves as a public safety-net hospital and level 1 trauma center.

This activity was approved by the UW human subjects division.

### 2.1 Algorithm 1 (ICD-based query)

Inpatient hospital billing data were used to identify and extract patients discharged with a primary or secondary diagnostic code for infective endocarditis within our clinical data warehouse using Microsoft SQL Server Management Studio (SQL). Selected codes were Ninth Revision (ICD-9) 424.9, 424.91, 424.99, 421.0, 421.1, 421.9, 112.81, 036.42, and Tenth Revision (ICD-10) I38, I39, I33, I33.9, B37.6, A39.51. This query was performed with and without a Current Procedural Terminology (CPT) code for echocardiogram (93303–93356).

### 2.2 Algorithm 2 (discharge summary-based query)

After conducting a review of thirty randomly selected discharge summaries (DS) over the period of interest, we identified four commonly employed patterns for discharge (or death) summary. From these, a key-word, pattern-based text query of discharge summaries (DS) was generated that selected on the term “endocarditis” in the fields headed by “Discharge Diagnosis” or “Admission Diagnosis” or “Other disease affecting hospitalization.” Patients containing these DS features were extracted from the data warehouse using SQL. Further coding involved the removal of possible “history of endocarditis” word combinations (e.g., “Hx of,” “Hx,” “H/O,” “history of”). Additional coding was performed to extract the nature and type of valve (prosthetic, tricuspid, pulmonic, aortic, or mitral), and the microbiologic agent responsible for the IE, if present in the diagnosis fields.

### 2.3 Case adjudication

DS query cases were chart reviewed by clinicians using a standardized collection form using REDCap electronic data capture hosted at the University of Washington.^[19]^ Four medical students conducted this first-pass review. The pre-specified criterion for a confirmed IE case was evidence of endocarditis mentioned but also verified within an infectious diseases (ID) consultation note. When patients did not have this ID consultation or verification, or had a clinical diagnosis of endocarditis but no evidence of valvular vegetation on transthoracic or transesophageal echocardiogram, charts were subsequently reviewed by two ID specialists, and cases were included in the cohort if they met Duke's criteria for definite IE (2 major or 1 major + 3 minor criteria).^[20]^ Interrater reliability was also assessed for a random 10% of all DS-identified student-reviewed cases with a secondary review by an ID specialist blinded to the original ascertainment; agreement with the first-pass review and kappa were calculated.^[21]^ Endocarditis cases that presented first from an outside facility were included as long as they continued to be treated for IE on transfer. Only incident cases of IE were included—such that if the patient was readmitted for the same endocarditis infection that was managed in an earlier hospitalization, only the earlier one was counted. Cardiovascular implantable electronic device infections were excluded unless they involved a valvular vegetation. Right-sided endocarditis was considered present in the absence of a tricuspid valve vegetation if there was septic pulmonary embolism in the absence of another embolic source.

Because we did not have a gold standard for case ascertainment of all possible cases of IE in our system that could be utilized without our screening methods, a separate set of IE cases was derived from two independent sources of data:

1. the HMC Infectious Diseases clinic's list of outpatient parenteral antibiotic therapy (OPAT) cases from 2016 to 2018 (n = 90) and
2. the Cardiology division's UWMC IE cases from their 2017 to 2018 echocardiogram lists (n = 100).

These were also adjudicated by an ID specialist to fulfill the same criteria as noted above for a final list of 166 confirmed IE cases. This cleaned list was used as a benchmark to evaluate the sensitivity of either algorithm. A list of 119 cases that were verified as non-IE cases from the HMC OPAT list was used to benchmark specificity.

ICD-identified cases (with or without CPT code) were assessed against the DS-identified and clinician-confirmed IE cases as well as the manually identified true IE cases for verified matches. All remaining ICD-screened cases that did not match these two confirmed lists of cases were then reviewed by an ID specialist using the adjudication criteria outlined above.

Baseline characteristics including age, sex, race, and ethnicity, as well as clinical comorbidities, were provided for descriptive purposes. Comorbidities were extracted using ICD-9/10 codes previously validated for Charlson comorbidities.^[22]^

### 2.4 Statistical analysis

The positive predictive value was calculated for each algorithm as the total number of verified/confirmed cases over the algorithm-selected cases. Sensitivity was calculated as the total number of algorithm-matched cases over the final list of 166 manually extracted true IE cases. Specificity was defined as the total number 119 non-cases minus the number of algorithm-matched cases over the 119 non-cases. Comparisons of these performance characteristics by algorithm were done with Chi-square testing. We calculated 95% Wilson score confidence intervals (CI)^[23]^ using R version 4.0.3.

## 3 Results

The ICD-based query identified 612 individuals with hospitalizations from July 1, 2015 to July 31, 2019 that included a billing code for infective endocarditis. Of these, 534 individuals also had an echocardiogram as part of this hospitalization. In contrast, for the same timeframe, the discharge summary-based query yielded 387 individuals. Baseline characteristics including age, sex, race, ethnicity, and comorbidities were similar between these groups (Table 1). Comparable to other endocarditis cohorts,^[1]^ the majority of patients were male.

**Table 1:** Baseline characteristics of two endocarditis cohorts, identified by diagnostic codes plus echocardiogram or by discharge summary.

Characteristic	ICD + ECHO n = 534		Discharge summary n = 387		*P*-value^a^
Age, median years (IQR)	47 (33, 63)		45 (32, 61)		.41
Female	185	35%	148	38%	.29
Race					.96
White	412	77%	299	77%	
Black or African American	53	10%	34	9%	
American Indian or Alaska Native	27	5%	21	5%	
Asian/Pacific Islander	31	6%	23	6%	
Unavailable or Unknown	11	2%	10	3%	
Ethnicity					.57
Hispanic or Latino	32	6%	17	4%	
Not Hispanic or Latino	487	91%	359	93%	
Unavailable or Unknown	15	3%	11	3%	
Comorbidities					
Any malignancy	91	17%	56	14%	.34
Cerebrovascular disease	222	42%	158	41%	.87
Chronic pulmonary disease	107	20%	66	17%	.29
Congestive heart failure	304	57%	236	61%	.24
Liver Disease	24	4%	19	5%	.89
Metastatic solid tumor	46	9%	17	4%	.02
Myocardial infarction	18	3%	13	3%	1.00
Peripheral vascular disease	126	24%	94	24%	.87
Renal disease	164	31%	120	31%	.98

ECHO = echocardiogram, ICD = International Classification of Diseases diagnostic code, IQR = interquartile range.

a *P*-values for comorbidities conducted per category instead of overall distribution since individual patients can have multiple comorbidities.

**Figure 1:**
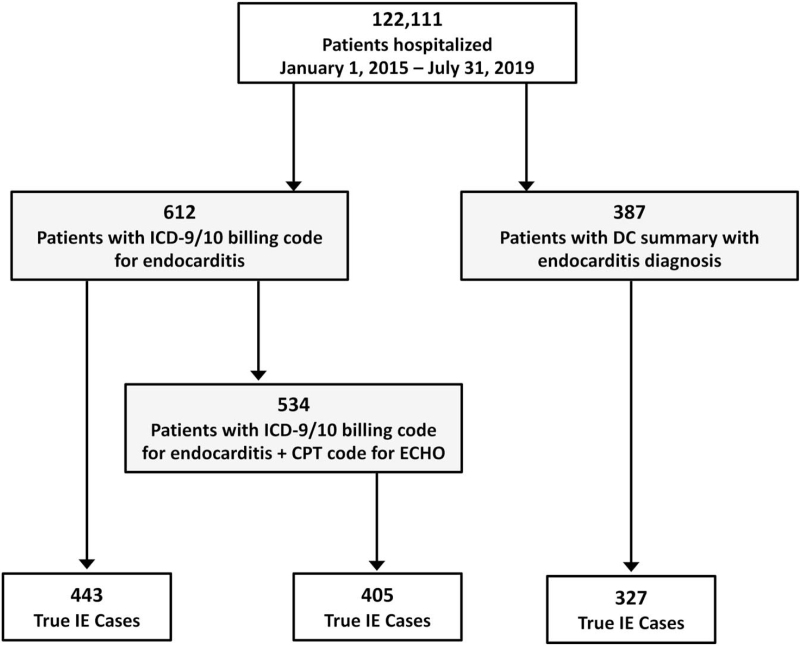
Flowchart of individuals identified through diagnostic codes (with or without Echocardiogram Procedure Codes) versus discharge summary.

Chart review of the 387 individuals identified through the DS query resulted in 327 patients with confirmed endocarditis. In contrast, the ICD code query resulted in only 443 true cases out of the 612 total identified; ICD code + ECHO resulted in 405 true cases out of 534 (Fig. 1).

The DS query demonstrated a higher positive predictive value (PPV) at 84.5% (95% CI 80.6%, 87.8%) compared with 72.4% (95% CI 68.7%, 75.8%) for ICD alone (*P* < .001) or 75.8% (95% CI 72.0%, 79.3%) with ICD + echocardiogram (*P* = .002, Table 2). The sensitivity of the DS algorithm was lower at 75.9% compared with 93.4% for ICD alone (*P* < .001) or 86.8% with ICD + echocardiogram (*P* = .017). Specificity was high for all algorithms at 94% to 98%.

**Table 2:** Test Characteristics of ICD-based versus discharge summary-based algorithms for endocarditis cohort identification.

	Positive predictive value 95% confidence interval	Sensitivity 95% confidence interval	Specificity 95% confidence interval
ICD code only	**72.4%** (443/612) 68.7%, 75.8%	**93.4%** (155/166) 88.5%, 96.3%	**94.1%** (112/119) 88.4%, 97.1%
ICD code + ECHO	**75.8%** (405/534) 72.0%, 79.3%	**86.8%** (144/166) 80.8%, 91.1%	**94.1%** (112/119) 88.4%, 97.1%
Discharge Summary	**84.5%** (327/387) 80.6%, 87.8%	**75.9%** (126/166) 68.9%, 81.8%	**98.3%** (117/119) 94.1%, 99.5%
*P*-value comparing ICD only vs Discharge summary	**<0.001**	**<0.001**	**0.174**
*P*-value comparing ICD + ECHO vs Discharge summary	**.002**	**.017**	**.174**

ECHO = echocardiogram, ICD = International Classification of Diseases.

A secondary review of student-reviewed cases by an ID specialist demonstrated excellent concordance with 97% agreement in assessments and a kappa of 0.78.

The DS query yielded information on valve involvement (prosthetic, tricuspid, pulmonic, aortic, or mitral) in 60% and/or the responsible organism in 73% of identified cases with an accuracy of 94% and 90%, respectively when assessed by chart review.

There were 78 true cases of IE missed by the DS query but captured by the ICD + ECHO query. Examination of a sample of 36 (46% of the 78) of these cases revealed three main reasons for lack of capture within the DS query:

1. the missed DS had a different pattern or formatting that resulted in the query not detecting the case,
2. “endocarditis” was not mentioned in the discharge diagnosis list (e.g., “bacteremia” was used instead) or
3. an incorrect spelling of “endocarditis” occurred.

An atypical pattern/format, typo or lack of mention of IE within the DS were the primary reasons encountered for misses in all of the cases reviewed.

## 4 Discussion

We evaluated the diagnostic performance of two different case identification methods for infective endocarditis (IE): a traditional query using ICD codes with and without CPT codes for echocardiogram compared with a key word, pattern-based search of discharge summaries. We found that the DS query had a higher PPV than the ICD query with the ability to identify the type and nature of valve and organism involved in a majority of cases, albeit with some loss of sensitivity. The ICD query had suboptimal PPV, even with the addition of echocardiogram codes. Both methods were reasonably specific.

Our ICD-based query was not as predictive for IE as previously reported,^[12]^ a reminder that the performance characteristics of ICD codes may not be generalizable across different healthcare systems. In our health system, coding was not able to distinguish between true cases and situations where endocarditis was considered but ultimately ruled out. Historical cases and hospitalizations that did not address the endocarditis as a current problem were often not appropriately coded. As with any complex clinical diagnosis, cases that were treated as probable endocarditis, that did not officially meet Dukes’ criteria for definite IE, may have been excluded in our adjudication but included in other settings. Our ascertainment criteria were not only stringent regarding the fidelity of diagnosis but the timeliness since our hope was to use this cohort for longitudinal assessment of clinical outcomes such as mortality, which requires an accurate anchoring to time of presentation. Using ICD in conjunction with a diagnostic procedure (or other defining data element) may be helpful in improving PPV and anchoring to the initial presentation.^[17,24]^ However, the inclusion of echocardiogram did not significantly increase the PPV of our ICD-based algorithm, nor differentiate the rule-out cases.

The higher PPV with the DC summary algorithm was not surprising. The query was designed to target the region of a clinician-generated document that outlined the key diagnoses of the hospitalization and was therefore intrinsically less likely to contain false positives. The logic of this coding was straightforward and replicable, and could be adapted for other conditions and scaled in a way that natural language processing may not. Narrative data also has the ability to move beyond the absence or presence of a condition to other more nuanced features such as timing, severity, and relationship to other factors.^[25]^

The loss in sensitivity for the DS query was notable and suggests that this mode of case identification may not be optimal when used for the purpose of comprehensive case capture (for example, when the intent is to determine incidence or prevalence). One option for streamlining case identification might be to start with ICD code query (to optimize sensitivity) and follow this with DS query to minimize chart review; we were unable to evaluate this combination method. The reduced sensitivity of the DS query was mainly attributable to atypical patterns or formats in the DC summary as well as unusual choice or spelling of words. These aberrations were sporadic in nature and unlikely to be associated with significant selection bias. Accurate and comprehensive retrieval of data from clinical documentation for secondary use could benefit from structured and consistent formats in notes, as well as features such as auto-filling of clinician-selected diagnoses or prepopulated options for data elements rather than free-form typing or transcription.^[26,27]^

Our study had some limitations. This was a single academic health system with two hospitals, and our findings may not be applicable to other settings. While multiple chart reviewers with different levels of training could have introduced variability in ascertainment, a second-pass review for accuracy suggested good concordance. Lastly, the manually extracted list of true cases that we used for benchmarking sensitivity was a not randomly selected sample of known cases but a convenience sample drawn from two specialty practices over a narrower period of time, so our estimate of sensitivity may have been influenced by the selected nature of these lists. A strength of our study was that we evaluated all identified candidates for case ascertainment with a sufficiently large sample to obtain greater precision around the PPV estimate. We also utilized a standardized verification criteria and explored missed cases to gain a better understanding of potential deficiencies in these strategies.

When compared to traditional ICD code-based queries, text-based queries have the potential to improve accuracy of IE case ascertainment and extract key clinical variables from the electronic medical record. Although such methods may come at a slight cost to sensitivity, they have the potential to quickly and accurately define a cohort of patients with complex clinical diagnoses such as IE and reduce the burden of clinical chart review.

## Acknowledgments

Authors would like to acknowledge Tanner N. Muggli, Jody Sharninghausen, Jordan M. Takasugi, and Ty J Tietjen for their assistance with the chart review for this study.

## Author contributions

**Conceptualization:** Hyang Nina Kim.

**Data curation:** Ayushi Gupta, Kristine Lan.

**Formal analysis:** Ayushi Gupta, Kristine Lan.

**Investigation:** Hyang Nina Kim, Ayushi Gupta, Kristine Lan, Jenell Stewart, Shireesha Dhanireddy, Maria A. Corcorran.

**Methodology:** Hyang Nina Kim, Ayushi Gupta, Kristine Lan.

**Resources:** Shireesha Dhanireddy.

**Supervision:** Hyang Nina Kim.

**Validation:** Hyang Nina Kim, Jenell Stewart, Maria A. Corcorran.

**Writing – original draft:** Hyang Nina Kim.

**Writing – review & editing:** Hyang Nina Kim, Ayushi Gupta, Kristine Lan, Jenell Stewart, Shireesha Dhanireddy, Maria A. Corcorran.
