# Diagnostic accuracy of ICD code versus discharge summary-based query for endocarditis cohort identification: Erratum

**DOI:** 10.1097/MD.0000000000028713

**Published:** 2022-01-28

In the article, “Diagnostic accuracy of ICD code versus discharge summary-based query for endocarditis cohort identification”,^[1]^ which appears in Volume 100, Issue 51 of *Medicine*, the comorbidities were incorrectly listed in Table 1. They have now been updated as seen in the below table. These to not affect the conclusions in the article.

**Table 1:** Baseline Characteristics of Two Endocarditis Cohorts, Identified by Diagnostic Codes plus Echocardiogram or by Discharge Summary.

Characteristic	ICD + ECHO n = 534		DC summary n = 387		*P*-value
Age, median years (IQR)	47 (33, 63)		45 (32, 61)		0.41
Female	185	35%	148	38%	0.29
Race					0.96
White	412	77%	299	77%	
Black or African American	53	10%	34	9%	
American Indian or Alaska Native	27	5%	21	5%	
Asian/Pacific Islander	31	6%	23	6%	
Unavailable or Unknown	11	2%	10	3%	
Ethnicity					0.57
Hispanic or Latino	32	6%	17	4%	
Not Hispanic or Latino	487	91%	359	93%	
Unavailable or Unknown	15	3%	11	3%	
Comorbidities					
Any malignancy	91	17%	56	14%	0.34
Cerebrovascular disease	222	42%	158	41%	0.87
Chronic pulmonary disease	107	20%	66	17%	0.29
Congestive heart failure	304	57%	236	61%	0.24
Liver Disease	24	4%	19	5%	0.89
Metastatic solid tumor	46	9%	17	4%	0.02
Myocardial infarction	18	3%	13	3%	1.00
Peripheral vascular disease	126	24%	94	24%	0.87
Renal disease	164	31%	120	31%	0.98

DC = discharge, ECHO = echocardiogram, ICD = International Classification of Diseases diagnostic code, IQR = interquartile range.

## References

[1] Kim HN Gupta A Lan K. Diagnostic accuracy of ICD code versus discharge summary-based query for endocarditis cohort identification. *Medicine*. 2021 100:e28354. 34941148 10.1097/MD.0000000000028354 PMC8702270

